# Dealing With Immunoglobulin Shortages: A Rationalization Plan From Evidence-Based and Data Collection

**DOI:** 10.3389/fpubh.2022.893770

**Published:** 2022-05-19

**Authors:** Gerard Solís-Díez, Marta Turu-Pedrola, Marta Roig-Izquierdo, Corinne Zara, Antoni Vallano, Caridad Pontes

**Affiliations:** ^1^Gerència del Medicament, Servei Català de la Salut, Generalitat de Catalunya, Barcelona, Spain; ^2^Departament de Farmacologia, de Terapèutica i de Toxicologia, Unitat Docent Parc Taulí, Facultat de Medicina, Universitat Autònoma de Barcelona, Barcelona, Spain

**Keywords:** immunogolobulins, clinical management, evidence-based medicine, real-world data, shortages

## Abstract

**Background:**

The demand and consumption of immunoglobulins (IgGs) are growing, and there are many difficulties in obtaining supplies. The aim of the study was to analyze the evolution of IgG consumption and cost over a decade, describe the measures implemented for clinical management in the context of regional public health system, and evaluate the initial impact of these measures.

**Methods:**

We performed a retrospective longitudinal study including patients of all public health systems in Catalonia. First, we analyzed data on consumption and cost of IgGs during a period between 1 January, 2010 and 31 December 2021. Second, we analyzed the impact of a set of regional measures in terms of annual consumption and cost of IgGs. Regional measures were based on rational evidence-based measures and computer registries. We compared the data of year before applying intervention measures (1 January and 31 December 2020) with data of year after applying clinical management interventions (1 January and 31 December 2021). In addition, detailed information on clinical indications of IgG use between 1 January and 31 December 2021 was collected.

**Results:**

Overall, in terms of population, the consumption of IgGs (g/1,000 inhabitants) increased from 40.4 in 2010 to 94.6 in 2021. The mean cost per patient increased from €10,930 in 2010 to €15,595 in 2021. After implementing the measures, the mean annual estimated consumption per patient in 2021 was statistically lower than the mean annual estimated consumption per patient in 2020 (mean difference −47 g, 95% CI −62.28 g, −31.72 g, *p* = 0.03). The mean annual estimated cost per patient in 2021 was also lower than the mean annual estimated cost per patient in 2020 (the mean difference was –€1,492, 95% CI –€2,132.12, –€851.88; *p* = 0.027). In 2021, according to evidence-based classification, 75.66% treatments were prescribed for a demonstrated therapeutic evidence-based indication, 12.17% for a developed therapeutic evidence-based indication, 4.66% for non-evidence-based therapeutic role indication, and 8.1% could not be classified because of lack of information.

**Conclusion:**

The annual consumption and cost of IgGs have grown steadily over the last decade in our regional public health system. After implementing a set of regional measures, the annual consumption of IgGs per patient and annual cost per patient decreased. However, the decrease has occurred in the context of the coronavirus disease 2019 (COVID-19) pandemic, which may have influenced their clinical use. Managing the use of IgGs through a rational plan with strategies including evidence-based and data collection may be useful in a shortage situation with growing demand. Registries play a key role in collection of systematic data to analyze, synthesize, and obtain valuable information for decision support. The action developed needs close monitoring in order to verify its effectiveness.

## Introduction

Cell blood components and plasma-derived medicinal products (PDMPs) are complex biologic therapies with multiple uses in immumodulation and replacement treatments of constitutional or acquired diseases of hemostasis (1–3). Some of these conditions are chronic, severe, or rare diseases, but PDMPs are also used in emerging medical situations and in medical management of acute conditions. Plasma allows obtaining a dozen of protein products such as albumin, polyvalent immunoglobulins (IgGs), coagulation factors, anti-proteases, and biologic adhesives (4). The plasma used for manufacturing medicinal products is recovered whole blood donated for transfusion purposes or collected by apheresis from donors in a proportions that varies depending on region and country (5).

Plasma for fractionation is considered a strategic resource, and plasma-derived medicines are considered essential medicines by the World Health Organization (WHO). The WHO has announced the implementation of a blood supply system founded on voluntary non-remunerated blood donation (VNRBD) and the establishment of not-for-profit blood establishments with the aim of achieving self-sufficiency in blood supply (6, 7). Furthermore, European union (EU) policies are driven by principles of voluntary unpaid donation of blood and blood components (8), although some countries have begun to compensate donors directly or indirectly (9).

The need for IgGs is currently the most important driver of the plasma fractionation industry. IgGs are required for the chronic treatment of patients with primary and secondary immunodeficiencies (PIDs and SIDs). In particular, SIDs create a growing demand, especially those derived from cancer chemotherapy and stem cell transplantation. They are also used as an immunomodulatory therapy for several disorders such as immune thrombocytopenia (ITP), Kawasaki disease, Guillain-Barré syndrome, chronic inflammatory demyelinating polyneuropathy (CIDP), and multifocal motor neuropathy, together with a large number of widely accepted off-label uses and other indications under investigation (10). Previous studies carried out in our local environment showed that intravenous IgGs were prescribed for a significant number of non-authorized and non-accepted indications with a notable cost, as well as important variability in intravenous IG (IVIG) prescriptions between hospitals (11). As the number of both label and off-label indications increases, and because most of these therapeutic uses are chronic in nature, the global demand for immunoglobulins is growing by about 6 to 8% per year (12).

In the last years, intermittent shortages due to limited supply of plasma to IgG producers are increasingly frequent. The stress in availability has translated into what has been described as “*the product follows the price*”, that is, difficulties in access to supplies in countries with lower acquisition prices (13). This is the case in Spain, where the reimbursement price for IgGs is among the lowest in terms of European prices (7). The COVID-19 pandemic has lowered surgical activity and donation, disrupting supply, and has increased product demand for investigational therapies for COVID-19, leading to further stress on the system (14).

Strategies to guarantee access to treatment with IgGs include long-term measures to achieve self-sufficiency of countries, increased donation of plasma, and improved manufacturing. On the other hand, the evidence of the efficacy and safety of IgGs is available only for a number of indications, with doubtful uses reported to represent up to 40% of total; thus, rational and balanced use of the available product is also required. If ever shortages lead to stock breakage, handling may require coordination and redistribution of the available product, avoiding stockpiling and prioritization to ensure that the available product covers patients who are at serious risk, avoiding use in weak off-label indications (10).

In Catalonia, the processing of plasma obtained from VNRBD in hospitals and blood donor campaigns is performed by a private vendor, and the resulting domestic immunoglobulin product is centrally managed by a public healthcare center (BST) owned by the Catalan Health Service. This organization has commissioned the integral management of these products, including a model for commercialization to hospitals at a fixed rate. According with interim data in 2021 BST offered up 30.7% of the Catalan immunoglobulin consumption, fixing as a self-sufficiency CHS rate.

In Catalonia, the Catalan Healthcare System (CHS) is integrated in a single network for public use of all healthcare resources with a variety of providers and management formulas. In 2000, the comprehensive health system for public use in Catalonia (SISCAT) was created, involving all different healthcare networks into a single public system. Some of the entities in the system are owned by the Department of Health or Catalan Health Services (CatSalut), public companies (public law entities subject to the private legal system and commercial companies), consortia, and foundations. SISCAT is currently composed of hospitals, primary care centers, social and health inpatient centers, in-patient mental health centers, basic and advanced life support ambulances (SVB and SVA), and the medical emergency systems such as non-urgent medical transport ambulances (rehabilitation, dialysis, etc.).

In June 2019, the CHS received several inputs on concerns from several hospitals regarding growing difficulties in obtaining immunoglobulin supplies. Posteriorly, Spanish Medicines shortage registry published firsts reports about punctual availability problems for non-specific IgGs medicines (15).

There are reported previous experiences of IgGs shortage in other countries, but health policy-makers did not respond to the shortage in a timely and effective manner. Those experiences indicated that shortages in IVIG would benefit from more immediate and coordinated efforts to make the distribution of IgGs in a just manner, and to identify and remedy the sources of IgG supply problems (16). However, no experiences have been reported on measures implemented to manage these situations of IgG supply problems from public health systems and the impact of these measures.

The aim of the study was to analyze the evolution of IgG consumption and cost over a decade, describe the contingency measures planned and implemented for clinical management in the context of public health system in Catalonia (CHS), and evaluate the initial impact of these measures.

## Materials and Methods

A retrospective longitudinal study including all patients of SISCAT was carried out. First, we analyzed data on consumption and cost of IgGs during a period between January 1, 2010 and December 31, 2021. Second, we analyzed the impact of the implementation of interventional measures in terms of annual consumption, doses, and cost of IgGs. We described and compared the data of the year before applying intervention measures (1 January and 31 December 2020) with data of the year after applying clinical management interventions (1 January and 31 December 2021). In addition, detailed information on clinic indications of IgGs use between 1 January and 31 December 2021 was collected.

Annual information about the following variables were recorded: number of patients, estimated annual consumption of IgGs (g), cost of IgGs (€), and type of IgGs classified as domestic IgG obtained from Catalan (VNRBD) and imported IgG obtained from remunerated blood donors (RBDs). This information was obtained from billing registry.

Since 2012, Catalonia has been implementing a dedicated registry, the Registry of Treatments and Patients (RPT), that includes detailed information on indications and use of hospital drugs for outpatient use, data on pragmatic assessments on effectiveness and safety are also collected for drugs that have been evaluated in the Catalan Pharmaco-Therapeutic Harmonization Program (17, 18). The registry is available for SISCAT hospitals, so all eligible drugs used in the public system and reimbursed by the Catalan Health Service must be registered for invoicing. Data can be cross-referenced to administrative databases, such as the Central Insurance Registry (RCA) and registries of billing, in order to extract aggregated reports on patient characteristics and economical costs for management purposes (19). As in 2019, the RPT was a suitable tool to collect both quantitative and qualitative detailed information on clinical use, and recorded information on IgG use in the registry was agreed upon by all hospital centers prescribing these products. Variables were chosen by consensus with hospital representatives and included therapeutic indication, administration route, dates of start and end of treatment, patient's weight, expected length of treatment and monthly dose (g/Kg), and required follow-up. Since August 2020, ongoing data collection and its inclusion in the RPT has been mandatory because of its link with invoicing of the product by the CHS.

A closed list of thirty-nine predetermined therapeutic indications was populated in the registry to cover most frequent uses (Supplementary Material). A free-text field was also enabled to register other different indications. Therapeutic indications were grouped with a dimension table according to different perspectives, such as overall therapeutic objective (replacement therapy in PID, replacement therapy in SID, immunomodulation, and solid organ or hematological transplantation) and established clinical adequacy according to evidence.

Periodic descriptive analysis, reporting of data, and methodology to do an assessment of clinical adequacy were agreed upon by consensus. For that purpose, three documents were taken into account that recommended or not the use of IgGs according to their degree of evidence and established clinical criteria for use (20–22). The clinical adequacy categorization method assigned the same evidence-category if three documents were consistent, and the lowest category if those were inconsistent.

Therapeutic indications were classified into five clinical use categories as follows:

- Demonstrated therapeutic role (category A): for this clinical indication, IgG use has reasonable-quality evidence support;
- Developed therapeutic role (category B): IgGs use is supported in selected patients with specific conditions, although the quality of evidence is variable;
- Exceptional therapeutic role (category C): only exceptionally would require IgG therapy, either because there are alternative therapies or because the evidence of benefit does not justify use in most cases;
- Non-evidenced therapeutic role (category D): there is evidence of no benefit, insufficient evidence of benefit, or some evidence of benefit but preferred alternative therapies are available;
- Non-classifiable: the category is used when information about a clinical indication narrowed down by a clinician was insufficient to be classified.

Besides all these, CatSalut distributed information to different SISCAT hospitals on the use of IgGs together with various recommendations for use in order to prioritize and optimize ongoing treatments.

- Optimal clinical management was recommended in order to concentrate the use of IgGs as much as possible on treatments with a higher degree of evidence, reconsidering the suitability of treatments with a low level of evidence. In addition, careful review of the use of IgGs in indications with a lower degree of evidence by analyzing the availability of other therapeutic alternatives in each of the indications is advised.
- Lists of patients with low-evidence indications of IgGs have been distributed to hospitals in order to facilitate case review and encourage prioritized use, adjusting to the current product availability
- Reassessment of diagnoses every 2-5 years. Diagnostic confirmation and periodic reassessment of the treatment requirement is advised in immunodeficiencies of adults without a molecular diagnosis confirming IgG deficiency.
- Adjustment of dosage by ideal weight in patients with indications of immunomodulation. In patients with a BMI> 30, the dosage may be reviewed to the adjusted ideal weight (40% by weight). In the dosage range adjusted for the doses of the presentations, in pediatric patients it may be adjusted upward, and in adult patients, it may be adjusted downward (23, 24).
- Individual evaluation of the interchangeability of presentations. Although this particular strategy cannot be applied systematically because of risks of associated adverse reactions, this option could be considered in the event of a supply failure (25, 26). It should be noted that there are patients who do not tolerate certain IgG brands, and those who tolerate must be maintained.
- Dose reduction and temporary treatment discontinuation in immunomodulatory indications when patients are in remission (10, 25, 27). This strategy has also been described as a feasible option for common variable immunodeficiency but is controversial without unanimous agreement (28).
- Available treatment alternative to IgG use is another available option (27). A document is being developed on potential therapeutic alternatives to IgGs in therapeutic indications where they are available and with solid evidence.

Since July 2021, the allocation of the proportion of the lots has been agreed to be based on the amount of use of IgGs for indications categorized as A and B. Likewise, criteria of minimum and territorial distribution is taken into account to ensure covering the needs of smaller centers and/or with special needs. A reserved amount is also allocated for emergencies. All territorial and emergency issues were overseen by the working group and agreed upon by consensus with hospital healthcare providers.

To know the existing stock, trends, and IgG requirements in SISCAT hospitals, a stock registry, initially used to manage supplies during the COVID-19 pandemic was, re-profiled. In June 2021, the registry became an IgG registry to inform weekly about available units. This information permits the establishment of categories to estimate the availability of IgGs by center, medicine, or administration route.

All these measures were discussed by dedicated committees involving hospital representatives (physicians, pharmacists, and managers) and members of the Catalan Health Service, and the need to closely monitor the use and availability of IgGs was agreed upon. A working plan was designed with the following actions: (1) collect systematic data about IgGs treatments in outpatients, further analyse it, and elaborate periodic reports; (2) set up a working group responsible of design select adequacy, utilization indicators and evidence-based measures for rational use and determine when each measure should be implemented, (3) disseminate data from periodic reports and main measure to involved centers aiming to enhance clinical revision, access priority, establish interchangeable and alternatives strategy and measure impact in consumption, and (4) coordinate and improve equitable access to the limited IgGs.

Information was presented to stakeholders with the aim of raising awareness, and data were disseminated at pharmacy services of SISCAT hospitals with the aim of supporting clinical review cases of treatments with categories C and D and of patients with dosages above product information recommendations.

A statistical analysis for categorical and continuous variables was conducted using frequencies, means, confidence intervals of 95% (95% CI), or standard deviations (SDs). Statistical mean differences were evaluated by Student *t*-test. A two-tailed significance was set at level.05. The statistical analysis was performed using SPSS 26 (IBM Corp. Armonk, NY, United States).

## Results

**Figure 1:**
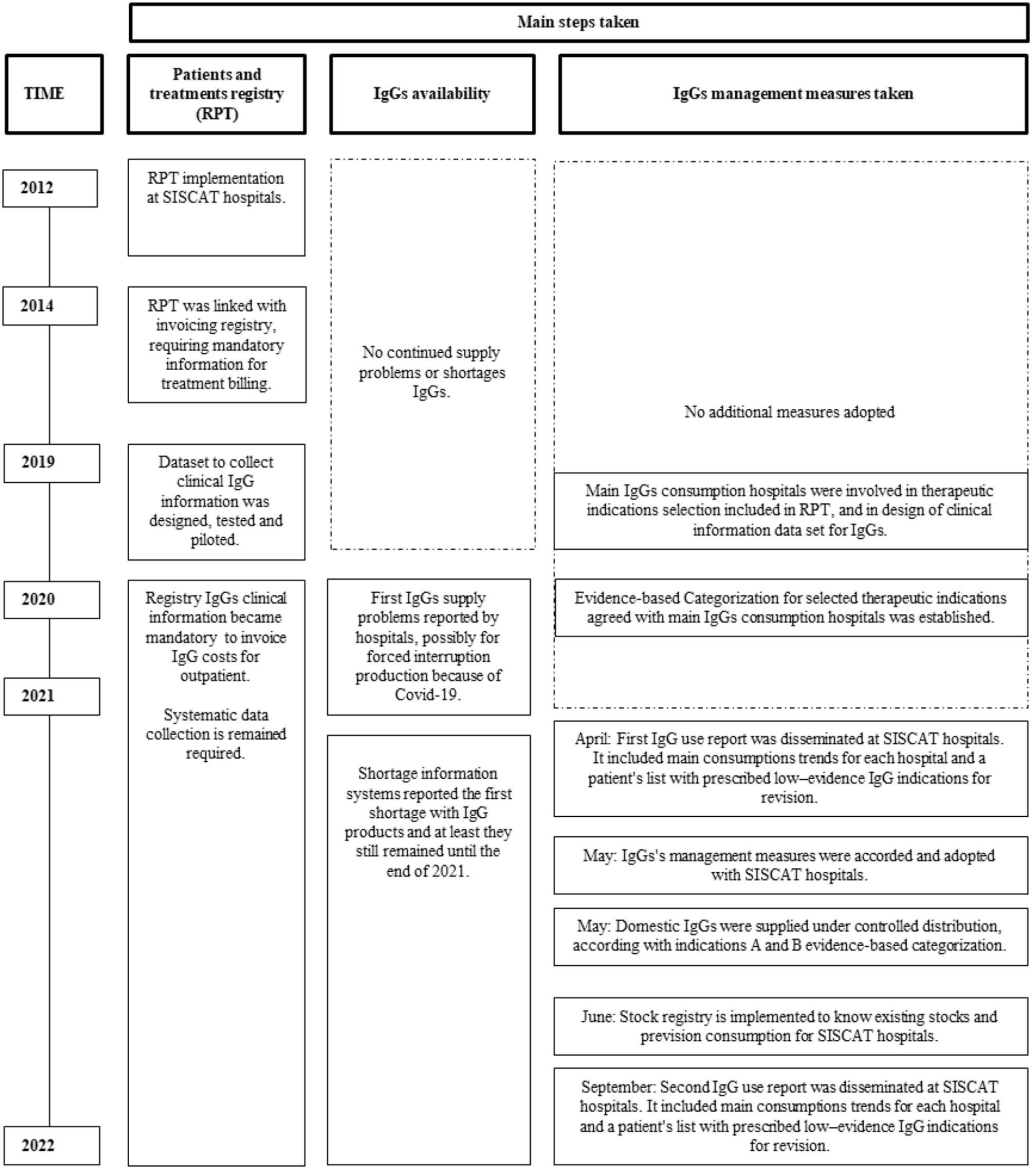
Main steps taken with patients and treatments registry, immunoglobulin (IgG) availability and IgG management measures taken trough time.

The main steps taken involving RPT, IgG availability, management measures, and trough time are summarized in Figure 1.

**Figure 2:**
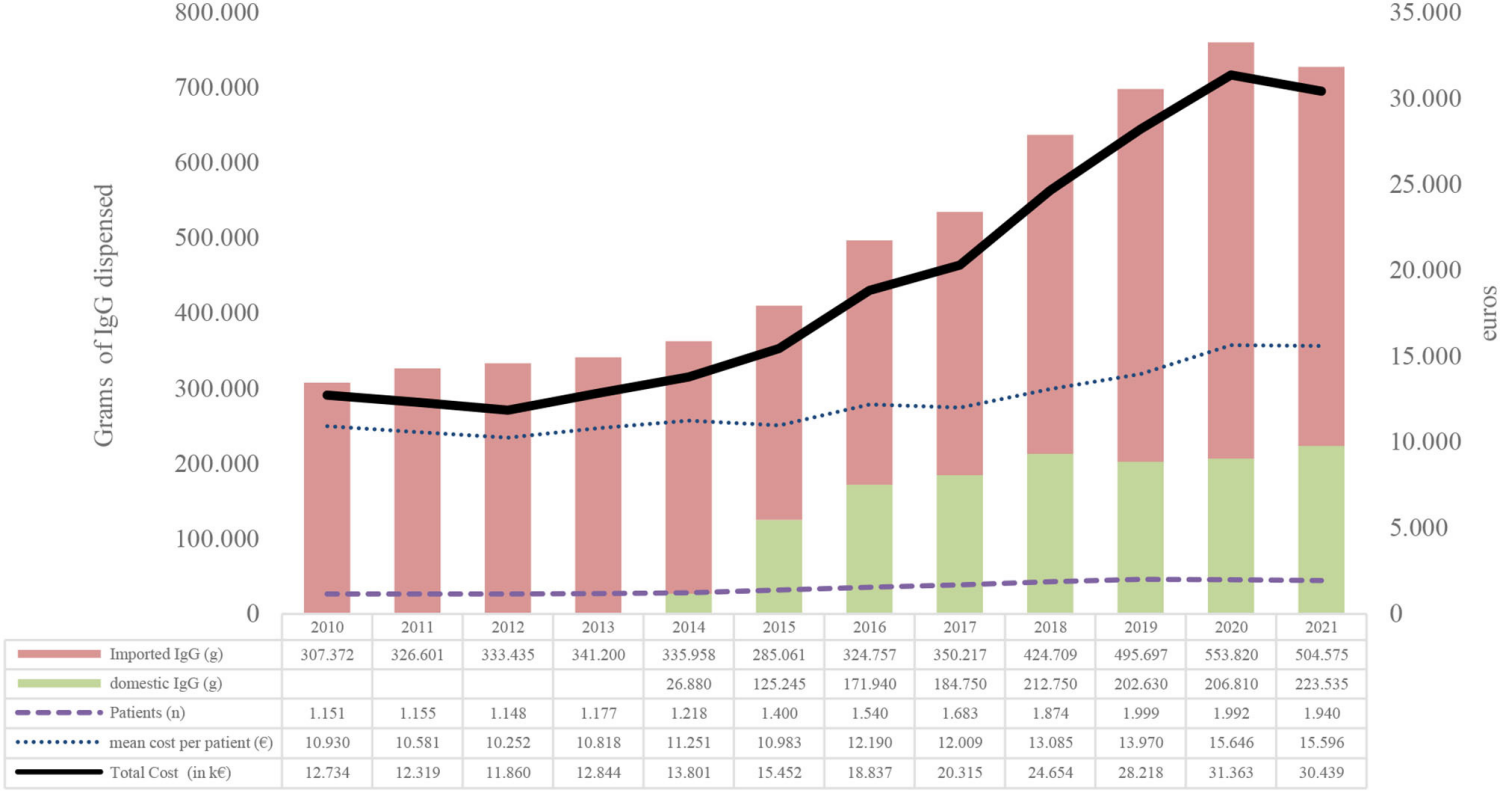
Evolution of IgG consumption between 2010 and 2021. Source, Billing registry;Domestic IgG, immunoglobulins obtained from Catalan voluntary non-remunerated blood donor (VNRBD); Imported IgG, immunoglobulins obtained from remunerated blood donors (RBDs).

Between 2010 and 2021, 5.94 million grams of non-specific IgGs were used for 6,925 patients at a cost of €232,835,146 and an average cost per patient in studied period of €33,391 (Figure 2).

In terms of population, the use in grams of IgGs for every 1,000 inhabitants has increased from 40.4 in 2010 to 94.6 in 2021, with an average annual increase of 7.3% (Supplementary Material).

The number of patients has grown annually from 1,151 in 2010 to 1,940 in 2021, with an annual average of 4,5%. The cost per patient-year was the most variable indicator, ranging from −3.29 to 7.4%. In the last 4 years, the increase has been positive and has grown by 6.24% on average (Figure 2).

A total of 1,956 outpatients have been registered with immunoglobulin therapy in year 2021. Mean age (SD) of the patients was 53.35 (22.1) years, and 1,007 (51.5%) were women with variability in age and sex distribution by therapeutic indications. According to the therapeutic objective of the treatment, replacement therapy in PID represented 620 (31.7%) treatments, replacement therapy in SID 299 (15.29%) treatments; and immunomodulation 392 (20.04%) treatments including multifocal motor neuropathy (MMN), CIDP, ITP, and Guillain-Barré syndrome, among others. In addition, 223 (11.4%) treatments were used for myasthenia gravis and 101 (5.16%) for desensitization therapy or rejection treatments in solid organ or bone marrow transplants (Table 1). According to the evidence-based classification consulted, 1,480 (75.66%) treatments were prescribed for a demonstrated therapeutic indication (category A), 224 (12.17%) treatments for a developed therapeutic indication (category B), 93 (4.66%) treatments for exceptional therapeutic or non-evidenced therapeutic role indication (categories C and D), and 157 (8.1%) treatments could not be classified because of lack of information (Supplementary Material).

**Table 1:** Description of IgG use in year 2021.

**Medical condition**	**Therapeutic area**	**Clinical adequacy category**	**Patients**	**Centers**	**Total estimated consumption**	**Mean (SD) monthly dose**	**Mean cost per patient**
			**N (%)**	**N (%)**	**Annual grams**	**g/Kg**	**€**
**Replacement therapy in primary immunodeficiencies diseases**			**620 (31, 7)**	**42 (87, 5)**	**228.856**	**0, 57 (0, 3)**	**15.568**
Congenital hypogammaglobulinemias	Hematology	B	54 (2, 76)	17 (35, 42)	16.256	0, 69 (0, 46)	
Common variable immunodeficiency disease (CVID)	Immunology	A	311 (15, 9)	35 (72, 92)	130.440	0, 59 (0, 25)	
IgG subclass deficiency (with recurrent infections)		B	102 (5, 21)	22 (45, 83)	34.935	0, 47 (0, 22)	
Unspecified primary antibodies deficiencies		C	69 (3, 53)	19 (39, 58)	25.917	0, 64 (0, 41)	
Agammaglobulinaemias		A	61 (3, 12)	12 (25)	15.405	0, 49 (0, 31)	
Severe combined immunodeficiency (SCID)		A	21 (1, 07)	12 (25)	5.275	0, 55 (0, 23)	
Replacement therapy in HIV/AIDS		D	2 (0, 1)	3 (6, 25)	630	0, 42 (0)	
**Immunomodulation**			**392 (20, 04)**	**42 (87, 5)**	**262.973**	**1, 68 (0, 69)**	**29.780**
ITP in specific circumstances (surgery, other therapy contraindicated, chronic ITP, concurrent risk factors)	Hematology	A	50 (2, 56)	15 (31, 25)	10.155	1, 51 (0, 84)	
ITP with life-threatening hemorrhage or potential lifethreatening hemorrhage		A	34 (1, 74)	14 (29, 17)	8.665	1, 64 (1, 05)	
Kawasaki disease	Immunology	A	4 (0, 2)	5 (10, 42)	295	2, 11 (1, 33)	
Chronic inflammatory demyelinating polyneuropathy (CIDP)	Neurology	A	205 (10, 48)	33 (68, 75)	168.276	1, 72 (0, 58)	
Multifocal motor neuropathy (MMN)		A	71 (3, 63)	17 (35, 42)	67.042	1, 69 (0, 45)	
Guillain–Barré syndrome (GBS)		A	23 (1, 18)	13 (27, 08)	6.080	1, 66 (0, 73)	
Chronic inflammatory demyelinating polyradiculoneuropathy		A	3 (0, 15)	4 (8, 33)	1.565	1, 41 (1, 11)	
Immune-mediated neuropathy (IMN)		A	2 (0, 1)	2 (4, 17)	895	2, 78 (-)	
**Other therapeutic objectives/diseases**			**388 (19, 84)**	**36 (75)**	**182.630**	**1, 69 (1, 06)**	**22.250**
Dermatomyositis (DM)	Dermatology	A	46 (2, 35)	18 (37, 5)	45.235	1, 77 (0, 5)	
Autoimmune haemolytic anemia (AIHA)	Hematology	B	4 (0, 2)	5 (10, 42)	1.885	1, 24 (1)	
Haemolytic disease of the newborn (HDN)		C	3 (0, 15)	2 (4, 17)	35	5, 28 (4, 11)	
Systemic lupus erythematosus (SLE)	Immunology	D	12 (0, 61)	8 (16, 67)	3.925	1, 28 (0, 74)	
ANCA-positive systemic necrotising vasculitis		B	11 (0, 56)	11 (22, 92)	8.338	1, 19 (0, 76)	
Sjögren's syndrome		C	3 (0, 15)	4 (8, 33)	2.380	1, 42 (0, 74)	
Susac syndrome		C	2 (0, 1)	2 (4, 17)	630	1, 26 (0, 26)	
Myasthenia gravis (MG)	Neurology	A	223 (11, 4)	30 (62, 5)	68.997	1, 61 (0, 92)	
Paraneoplasic encephalitis		C	25 (1, 28)	13 (27, 08)	8.450	2, 35 (2)	
Inflammatory myopathies		A	16 (0, 82)	10 (20, 83)	14.300	1, 63 (0, 51)	
Lambert–Eaton myasthenic syndrome (LEMS)		A	12 (0, 61)	7 (14, 58)	5.250	1, 67 (0, 64)	
IgM paraproteinaemic demyelinating neuropathy		B	9 (0, 46)	8 (16, 67)	9.215	1, 92 (0, 59)	
Stiff person syndrome		A	9 (0, 46)	7 (14, 58)	5.060	1, 61 (0, 43)	
IgG and IgA paraproteinaemic demyelinating neuropathies		A	8 (0, 41)	7 (14, 58)	6.610	1, 38 (0, 46)	
Epilepsy		C	5 (0, 26)	4 (8, 33)	2.320	1, 54 (0, 53)	
**Replacement therapy in secondary immunodeficiencies diseases**			**299 (15, 29)**	**34 (70, 83)**	**61.362**	**0, 54 (0, 51)**	**8.910**
Replacement therapy in chronic lymphocytic leukemia and severe recurrent infections	Hematology	A	94 (4, 81)	22 (45, 83)	16.050	0, 4 (0, 14)	
Replacement therapy in multiple myeloma and severe recurrent infections		A	50 (2, 56)	10 (20, 83)	7.245	0, 49 (0, 52)	
Replacement therapy in chronic lymphocytic leukemia and recurrent infections		A	2 (0, 1)	3 (6, 25)	145	0, 38 (0, 15)	
Secondary hypogammaglobulinaemia (excluding hematological malignancies)	Immunology	B	127 (6, 49)	22 (45, 83)	28.994	0, 66 (0, 67)	
Replacement therapy patients with severe or recurrent infections, ineffective antimicrobial treatment and either proven specific antibody failure (PSAF)		B	26 (1, 33)	17 (35, 42)	8.928	0, 56 (0, 2)	
**Transplantation related therapy**			**101 (5, 16)**	**11 (22, 92)**	**12.451**	**0, 75 (0, 52)**	**5.397**
Replacement therapy pre or post allogenic stem cell transplantation with hypogammaglobulienemia	Transplant Medicine	B	48 (2, 45)	6 (12, 5)	5.046	0, 48 (0, 19)	
Treatment of antibody mediated solid organ transplant rejection		A	37 (1, 89)	10 (20, 83)	4.955	0, 93 (0, 54)	
Ongoing desensitization of patients to improve the likelihood of non-compatible ABO or HLA transplantation		A	13 (0, 66)	3 (6, 25)	2.100	1, 22 (0, 69)	
Ongoing desensitization of patients to improve the likelihood of solid transplantation with highimmunologic risk		A	3 (0, 15)	2 (4, 17)	350	1, 44 (0, 54)	
**Non-classifiable**		**E**	**156 (7, 98)**	**26 (54, 17)**	**67.979**	**1, 41 (1, 02)**	**18.812**
**Total**			**1956 (100)**	**48 (100)**	**816.251**	**1, 07 (0, 87)**	**18.305**

*A, Demonstrated therapeutic role; B, Developed therapeutic role; C, Exceptional therapeutic role; D, Non-evidenced therapeutic role (category D); E, Non-classifiable*.

*Bold values are superior categories that aggregate several indications showed below*.

Prescription was for a chronic pathology (≥ 1 year of treatment) for 1,563 (79.91%) patients. The mean (SD) of the estimated initial monthly dose was 1.07 (0.87) g/Kg per month. The mean of estimated annual consumption per patient was 435 (483.7) g of non-specific IgGs. According to the therapeutic objective of the immunoglobulin prescription, the mean (SD) of the monthly dose of IgGs was highest for diseases not related with replacement therapy or immunomodulation, which stood at 1.69 (1.06) g/Kg, followed by immunomodulation with 1.68 (0.69) g/Kg and other non-classifiable indications due to lack of information, with 1.41 (1.02) g/Kg (Supplementary Material).

Overall, the mean annual estimated consumption per patient (g) in 2021 was statistically lower than the mean annual estimated consumption per patient in 2020 (mean difference −47 g.; 95% CI −62.28; −31.72; *p* =0.03). In addition, the mean annual estimated cost per patient (€) in 2021 was also lower than the annual mean annual estimated cost per patient in 2020 (mean difference –€1,492; 95% CI, −2,132.12; −851.88; *p* = 0.027) (Table 2).

**Table 2:** Main differences between 2020 and 2021 trends in IgG consumption.

**Therapeutic objective**	**Key indicator**	**Year 2020**	**Year 2021**	**Mean differences (CI95%)**	***p*-value**
		**mean (CI95%)**	**mean (CI95%)**		
**Replacement therapy in primary immunodeficiencies diseases**	Number of patients	665	620		
	Annual estimated consumption (g)	402 (382.62; 421.38)	374 (356.53; 391.47)	−28 (−40.71;−15.29)	0.037
	Monthly dose (g/Kg)	0.62 (0.6; 0.64)	0.57 (0.55; 0.59)	−0.05 (−0.07;−0.03)	0.003
	Estimated annual cost (€)	16,722 (15,910.12; 17,533.88)	15,533 (14,796.71; 16,269.29)	−1189 (−1722.71;−655.29)	0.106
**Immunomodulation**	Number of patients	434	392		
	Annual estimated consumption (g)	785 (708.23; 861.77)	704 (630.75; 777.25)	−81 (−132.69;−29.31)	0.1
	Monthly dose (g/Kg)	1.74 (1.68; 1.8)	1.68 (1.61; 1.75)	−0.06 (−0.1;−0.02)	0.202
	Estimated annual cost (€)	32,149 (28,968.58; 35,329.42)	30,188 (27,052.69; 33,323.31)	−1,961 (−4,132.87;210.87)	0.39
**Other therapeutic objectives/diseases**	Number of patients	414	388		
	Annual estimated consumption (g)	558 (509.07; 606.93)	510 (460.35; 559.65)	−48 (−81.81;−14.19)	0.029
	Monthly dose (g/Kg)	1.7 (1.56; 1.84)	1.69 (1.58; 1.8)	−0.01 (−0.1;0.08)	0.913
	Estimated annual cost (€)	22,813 (20,790.04; 24,835.96)	22,547 (20,387.11; 24,706.89)	−266 (−1,698.85;1,166.85)	0.86
**Replacement therapy in secondary immunodeficiencies diseases**	Number of patients	274	299		
	Annual estimated consumption (g)	236 (208.17; 263.83)	211 (184.59; 237.41)	−25 (−43.59;−6.41)	0.202
	Monthly dose (g/Kg)	0.56 (0.5; 0.62)	0.54 (0.48; 0.6)	−0.02 (−0.06;0.02)	0.646
	Estimated annual cost (€)	9,745 (8,576.22; 10,913.78)	8,759 (7,647.97; 9,870.03)	−986 (−1,767.39;−204.61)	0.231
**Transplantation related therapy**	Number of patients	118	101		
	Annual estimated consumption (g)	137 (104.7; 169.3)	131 (104.28; 157.72)	−6 (−26.73;14.73)	0.784
	Monthly dose (g/Kg)	0.83 (0.7; 0.96)	0.75 (0.65; 0.85)	−0.08 (−0.16;0)	0.345
	Estimated annual cost (€)	5,776 (4,402.03; 7,149.97)	5,483 (4,327.29; 6,638.71)	−293 (−1,180.07;594.07)	0.754
**Non-classifiable**	Number of patients	129	156		
	Annual estimated consumption (g)	507 (399.84; 614.16)	453 (372.5; 533.5)	−54 (−117.81;9.81)	0.422
	Monthly dose (g/Kg)	1.42 (1.26; 1.58)	1.41 (1.25; 1.57)	−0.01 (−0.12;0.1)	0.931
	Estimated annual cost (€)	20,834 (16,312.62; 25,355.38)	18,542 (15,233.13; 21,850.87)	−2,292 (−4,953.04;369.04)	0.409
**All therapeutic objectives**	Number of patients	2034	1956		
	Annual estimated consumption (g)	483 (459.97; 506.03)	436 (414.55; 457.45)	−47 (−62.28;−31.72)	0.03
	Monthly dose (g/Kg)	1.1 (1.06; 1.14)	1.07 (1.03; 1.11)	−0.03 (−0.06;0)	0.301
	Estimated annual cost (€)	19,890 (18,935.35; 20,844.65)	18,398 (17,487.96; 19,308.04)	−1,492 (−2,132.12;−851.88)	0.027

By therapeutic objective, the mean annual estimated consumption per patient (g) in PID statistically decreased in 2021 (mean difference −28 g.; 95% CI: −40.71; −15.29; *p* = 0.037), and the mean monthly dose per patient in terms of g/kg (mean difference −0.05; 95% CI: = 0.003). The mean annual estimated consumption per patient in other objectives, mainly due to myasthenia gravis, was also statistically lower in 2021 (mean difference −48 g.; 95% CI: −81.81; −14.19; *p* 0 0.029) (Table 2).

Since stock registry was ongoing, median estimated availability was 19.7 days per month for IVIG and 25 days per month for subcutaneous IgGs (SCIG) (Supplementary Material).

## Discussion

There has been a sustained and continued increase in the demand for IgGs in recent years. A review looked at the causes of the large increase in demand for immunoglobulins by investigating guidelines, recommendations, Cochrane data analyses, and systematic reviews for clinical indications of IgGs over time. No new evidence explaining the huge increase was found, and market profitability seems to be the dominant driving force that explains this large increase in demand. This situation is likely to continue in the absence of good clinical studies analyzing the place of IgG therapy vs. other treatments (10).

Product shortages during the COVID-19 pandemic caused sudden increases in demand or disruptions in supply chains of different products such as IgGs. Experiences describe how health systems have handled shortages in essential products by estimating the clinical demand, assessing product supply and potential inequities in access, and enacting policies grounded in an ethical framework for drug allocation (29, 30).

In a shortage situation, contingency planning includes prioritization policies for patients in the event of predicted shortage. A range of strategies tries to maintain ongoing equitable access to blood for transfusion, sharing experience, and developing expert consensus on the basis of evolving publications, have all been proposed as useful tools in helping transfusion services and hospitals (31). Globally, national and regional health systems have designed IgG shortage management plans in last years to deal with possible critical scenarios (32–34). All agents must be implicated in the importance of the good clinical use of IgGs, in particular, and cell blood components and PDMP in general. This limited resource is vital in many medical areas, and major conclusions should be established to assess their clinical use.

The categories of adequacy were agreed upon as a surrogate of appropriate use, so the proportion of prescription in category A could be used as an element to prioritize access to available stocks whenever coordinated distribution was advised.

Regarding dose optimization, use of IgG dose adjusted for ideal body weight (IBW) is a desired option, since biological drugs are mainly distributed to the central compartment; thus, it is sensible to account for the excess of poorly perfused adipose tissue when body-weight adjustment of dosing is done. This is not only rational but also helps saving significant amounts of IgGs. The main guidelines for clinical use of IgGs include recommendations for the use of IBW in the calculation of the dosage weight (DW) of immunoglobulins. In all of them, a compromise is reached between current weight (CW) and IBW to determine the DW (23, 24). The working group is also developing guidance on therapeutic alternatives to IgGs in therapeutic indications where they are available and with solid evidence (24).

Medicine regulatory agencies have developed national shortage registries, but they generally inform once the supply has been compromised instead of early detecting misbalances between use and supply in real-time, thus predicting shortages and allowing early action to avoid or manage them (12).

One of the first proposals of the group was to prospectively collect information about therapeutic indications for which IgGs were prescribed, as well as dosages and posology, in order to analyze clinical adequacy and potential areas of improvement and efficiency.

Data availability on the use of IgGs from clinical records is a useful source of information on how to manage the use of IgGs. In that sense, therapeutic registries may also be useful, since they can approach effectiveness and utilization of registered medicines by collecting information on indication and, eventually, results. A benefit of this information when it is rapidly transformed into reports is in helping decision-making in health. However, an important finding was the consumption growth of IgGs in non-classifiable therapeutic indications. Therefore, more actions should be designed and implemented in order to improve the quality of recorded information.

A very important aspect is the variability observed between the centers in patterns of use of non-specific domestic IgGs and patterns of clinical use of therapeutic indication as in dosages, as well as economic expense. Therefore, a key issue is addressing the variability of usage. Measures for the dissemination of this information among the centers for benchmarking allow identifying and sharing best practices aimed to improve IgG use. Finally, it is also useful to establish common proposals and recommendations for all the centers to reduce the variability. Thus, it is very important to establish good communication with all centers, and to achieve the participation and co-responsibility of the centers in the management of the use of IgGs.

In a situation of scarcity, the concept of using biological agents at the lowest effective dose is logical and may help minimize side effects, some of which may be dose-related. This also saves significant amounts of IgGs. The use of immunoglobulin dose adjusted for ideal body weight (IBW) may be a rational approach.

In our experience, managing the use of IgGs through multiple strategies may be useful; however, we must take into account that observed reduction in the use of IgGs is also coincident with the pandemic period where other circumstances may have influenced their clinical use. Either increased use for COVID-19 related conditions or reduced use due to difficulties in healthcare access during epidemic waves have disrupt IgG consumption. The effectiveness of these policies should be appraised to know their impact on the heterogeneity of their availability, their use, and their clinical outcomes in patients treated with IgGs; furthermore, less consumption should be observed. In addition, shortage alert systems should be improved providing information in real-time and predicting shortage trends and if this manner could advance at critical situations and prepare responses with enough time. The actions developed need close follow-up. Continuous monitoring of proposed indicators is needed in the near future to verify its effectiveness.

Different studies on interventions to reduce low-value care have been carried out. However, little is known about how the effects of such interventions are measured. Most published studies were focused on reduction in utilization. The effectiveness of these interventions have been variable (35). Audit and feedback, computerized advice, point-of-care reminders, practice facilitation, educational outreach, and processes for patient review and follow-up all demonstrated evidence of a quality improvement effect. Evidence of an improvement effect was higher where baseline performance was low and was particularly demonstrated across process measures and measures related to prescribing. Evidence has not been sufficient to suggest that multifaceted approaches were more effective than single interventions (36). However, no study reports the cost-benefit ratio of documented interventions. Future intervention studies should incorporate economic variables to documents and disseminate the cost-benefit ratio.

The main limitations in analyzing the effect of the intervention were related to the study period and the lack of a control group. On the one hand, the study period coincided with the COVID-19 pandemic. Therefore, the main limitation was related to the effect of this disease, which has had a great influence on the observed results. On the other hand, the data are observational and are limited to comparing two periods with a very short time interval; therefore, it is difficult to analyze the effect in such a short time. A concurrent control group was not included to be able to analyze the causal relationship between the intervention and the observed effect. Although different health systems have developed evidence-based management plans for situations of immunoglobulin deficiency, there are no descriptions of the effect of these management plans. Therefore, it should be noted as strength that an initial experience has been presented on the effect of the implementation of specific measures for the clinical management of IgGs in the context of a regional health system. Nevertheless, a long-term follow-up is necessary to verify the effectiveness of these measures.

## Conclusions

The annual consumption and cost of IgGs increased in the last decade. After identifying a potential serious health problem consisting of increasing supply problems of IgGs, a set of pharmaceutical policies has been drawn up in conjunction with stakeholders. A multifaceted intervention based on evidence focused on assessing the clinical adequacy of actual treatments, optimizing indications and dosing, to ensure an equitable supply in centers was done. We used previous records and set up new *ad hoc* registries to evaluate the effect of this intervention on the use of IgGs. In a short period after the intervention, we observed a decrease in consumption of IgGs and in cost per patient but in the context of COVID-19. Therefore, long-term follow-up would be needed to better analyse the impact of the established measures. Registries play a key role in collection of systematic data to analyze, synthetize, and extract valuable information for decision support. More research is required to determine the use and impact of quality improvement interventions using theoretical frameworks and by cost-effectiveness analysis.

## Author Contributions

CP, AV, and GS-D conceptualized and designed the article and verified data. GS-D and MT-P collected the data, analyzed the data, and created the tables and graphs. GS-D drafted and wrote the manuscript. MR-I, CZ, MT-P, and AV reviewed the manuscript for important intellectual content. CP revised the final version of the manuscript and is guarantor for the article and expert in public health. All authors contributed to the article and approved the submitted version.

## Conflict of Interest

The authors declare that the research was conducted in the absence of any commercial or financial relationships that could be construed as a potential conflict of interest.

## Publisher's Note

All claims expressed in this article are solely those of the authors and do not necessarily represent those of their affiliated organizations, or those of the publisher, the editors and the reviewers. Any product that may be evaluated in this article, or claim that may be made by its manufacturer, is not guaranteed or endorsed by the publisher.
