# Supplementary material for: Dealing With Immunoglobulin Shortages: A Rationalization Plan From Evidence-Based and Data Collection
Source: Front Public Health. 2022 May 19;10:893770. doi: 10.3389/fpubh.2022.893770 (PMC9160570; doi:10.3389/fpubh.2022.893770)
Supplement: Supplementary file 1 [file Data_Sheet_1.pdf]

Supplementary Material

Supplementary Figure 1. Grams of IgG dispensed per 1,000 inhabitants in study period

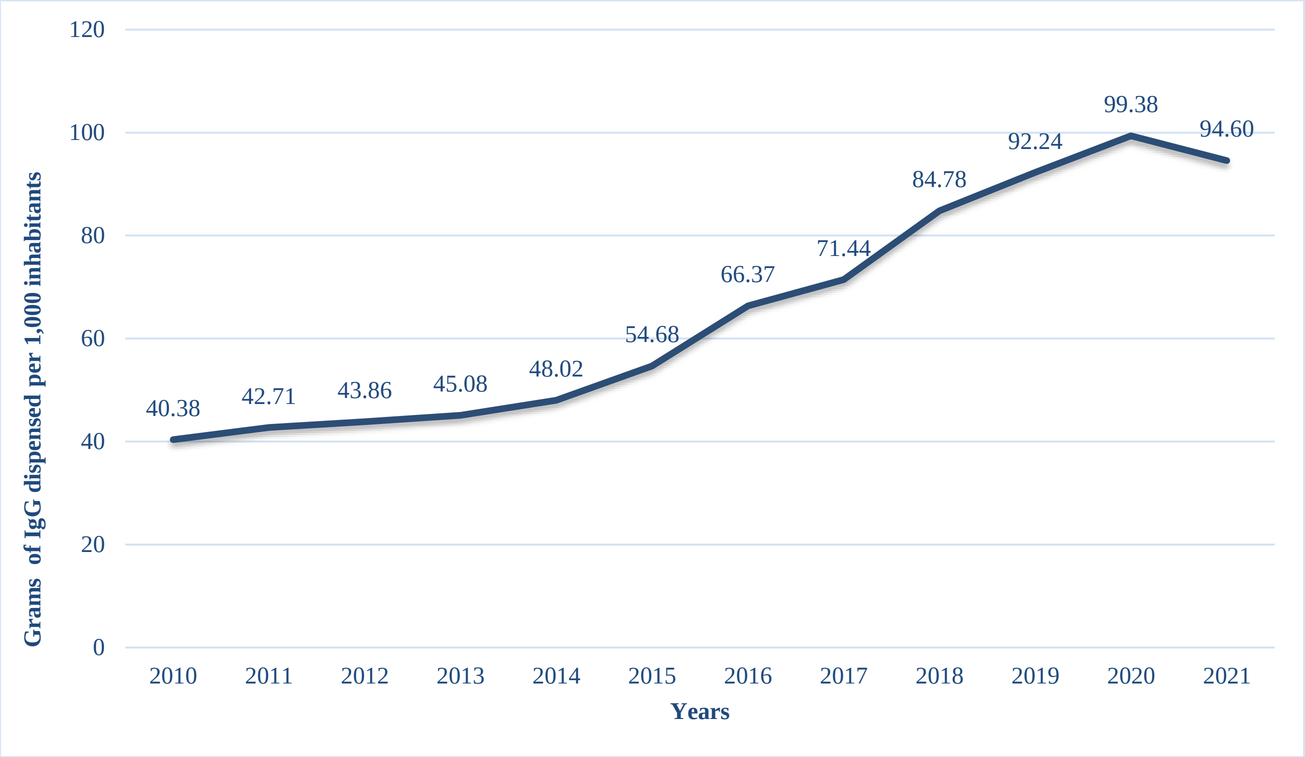

Source: Billing registry

Supplementary Table 1. Description of demographic characteristics by clinical adequacy categories and total (year 2,021).

	**A**	**B**	**C**	**D**	**E**	**Total**
Patients – n	1,480	224	91	2	159	**1,956**
Mean (SD) age in years	54.32 (21.5)	47.96 (25.96)	51.01 (23.09)	53.74 (39.08)	53.27 (19.3)	**53.35 (22.06)**
Women – n (%)	739 (49.93%)	139 (62.05%)	41 (45.05%)	(0%)	88 (55.35%)	**1007 (51.48%)**
Chronic treatment (≥1 year)	1193 (80.61%)	179 (79.91%)	75 (82.42%)	2 (100%)	114 (71.7%)	**1563 (79.91%)**
Total amount IgG grams dispensed in g	667,595.50	71,134.50	42,566.50	630.00	70,809.00	**852,735.50**
Mean (SD) monthly dose (g/Kg)	1.05 (0.78)	1.01 (1.06)	1.01 (1.18)	0.42 (0)	1.4 (1.02)	**1.07 (0.87)**

Supplementary Table 2. Therapeutic indications reported and assessment of evidence

**Medical condition**	**Therapeutic objective**	**Therapeutic Area**	**Clinical adequacy***
Congenital hypogammaglobulinemias	Replacement therapy in primary immunodeficiencies diseases	Haematology	B
Common variable immunodeficiency disease (CVID)		Immunology	A
IgG subclass deficiency (with recurrent infections)		Immunology	B
Unspecified primary antibodies deficiencies		Immunology	C
Agammaglobulinaemias		Immunology	A
Severe combined immunodeficiency (SCID)		Immunology	A
Replacement therapy in HIV/AIDS		Immunology	D
ITP in specific circumstances (surgery, other therapy contraindicated, chronic ITP, concurrent risk factors)	Immunomodulation	Haematology	A
ITP with life-threatening haemorrhage or potential lifethreatening haemorrhage		Haematology	A
Kawasaki disease		Immunology	A
Chronic inflammatory demyelinating polyneuropathy (CIDP)		Neurology	A
Multifocal motor neuropathy (MMN)		Neurology	A
Guillain–Barré syndrome (GBS)		Neurology	A
Chronic Inflammatory Demyelinating Polyradiculoneuropathy		Neurology	A
immune-mediated neuropathy (IMN)		Neurology	A
Dermatomyositis (DM)	Other therapeutic objectives/diseases	Dermatology	A
Autoimmune haemolytic anaemia (AIHA)		Haematology	B
Haemolytic disease of the newborn (HDN)		Haematology	C
Systemic lupus erythematosus (SLE)		Immunology	D
ANCA-positive systemic necrotising vasculitis		Immunology	B
Sjögren’s syndrome		Immunology	C
Susac syndrome		Immunology	C
Myasthenia gravis (MG)		Neurology	A
Paraneoplasic encephalitis		Neurology	C
Inflammatory myopathies		Neurology	A
Lambert–Eaton myasthenic syndrome (LEMS)		Neurology	A
IgM paraproteinaemic demyelinating neuropathy		Neurology	B
Stiff person syndrome		Neurology	A
IgG and IgA paraproteinaemic demyelinating neuropathies		Neurology	A
Epilepsy		Neurology	C
Replacement therapy in chronic lymphocytic leukaemia and severe recurrent infections	Replacement therapy in secondary immunodeficiencies diseases	Haematology	A
Replacement therapy in multiple myeloma and severe recurrent infections		Haematology	A
Replacement therapy in chronic lymphocytic leukaemia and recurrent infections		Haematology	A
Secondary hypogammaglobulinaemia (excluding haematological malignancies)		Immunology	B
Replacement therapy patients with severe or recurrent infections, ineffective antimicrobial treatment and either proven specific antibody failure (PSAF)		Immunology	B
Replacement therapy pre or post allogenic stem cell transplantion with hypogammaglobulienemia	Transplantation related therapy	Transplant Medicine	B
Treatment of antibody mediated solid organ transplant rejection		Transplant Medicine	A
Ongoing desensitisation of patients to improve the likelihood of non-compatible ABO or HLA transplantation		Transplant Medicine	A
Ongoing desensitisation of patients to improve the likelihood of solid transplantation with high immunologic risk		Transplant Medicine	A
Non-classifiable		Transplant Medicine	E

*According to available evidence

Supplementary Figure 2. Median monthly dose (g/Kg) by therapeutic objective in active patients (year 2020). 2A) Replacement therapy in primary immunodeficiencies (PID). 2B) Replacement therapy in secondary immunodeficiencies. 2C) Immunomodulation therapy; 2D) Other indications

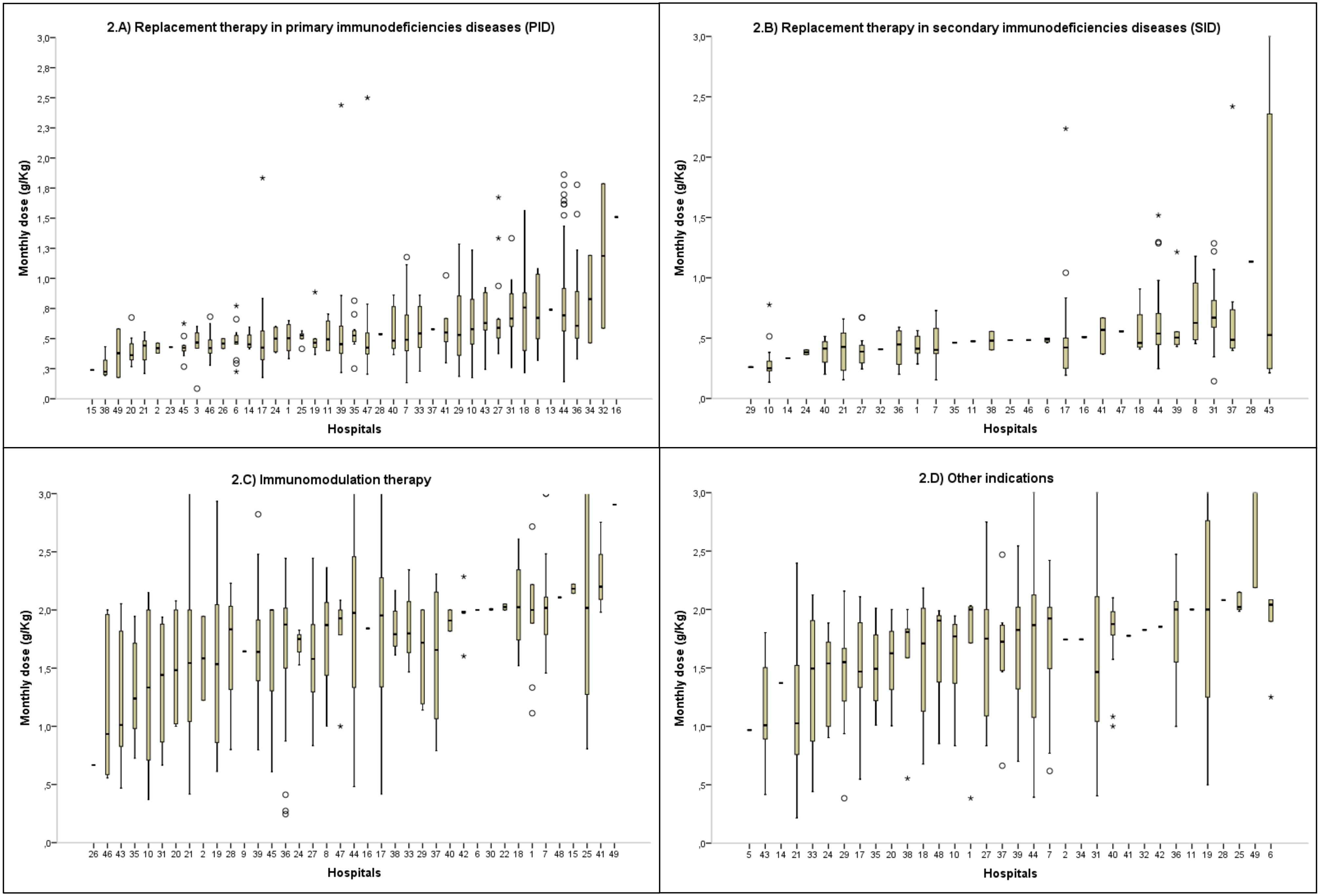

Supplementary Table 3. Expected monthly of IgG coverage for SISCAT Hospitals (n = 48)

	**July 21**		**August-21**		**September-21**		**October-21**		**November-21**		**December-21**	
	**monthly stock (g)**	**daily consumption (g)**	**monthly stock (g)**	**daily consumption (g)**	**monthly stock (g)**	**daily consumption (g)**	**monthly stock (g)**	**daily consumption (g)**	**monthly stock (g)**	**daily consumption (g)**	**monthly stock (g)**	**daily consumption (g)**
**IVIG***	30.512,5	1.811,3	44.667,5	2.921,8	45.428,5	1.774,9	55.189,0	3.410,1	61.273,0	2.627,4	55.656,5	2.166,4
**SCIG****	3.338,0	186,0	4.539,0	215,9	3.637,0	103,1	6.804,0	226,2	6.616,0	181,0	4.295,0	146,8
	**expected coverage days per month**		**expected coverage days per month**		**expected coverage days per month**		**expected coverage days per month**		**expected coverage days per month**		**expected coverage days per month**	
**IVIG**	17,9		21,0		35,3		30,1		36,5		29,3	
**SCIG**	16,8		15,3		25,6		16,2		23,3		25,7	
*IVIG: intravenous immunoglobulin; **SCIG: Subcutaneous immunoglobulin												
